# Spatial modeling of soil-transmitted helminthiases in Colombia under climate change scenarios

**DOI:** 10.7705/biomedica.7965

**Published:** 2025-11-27

**Authors:** Mario J Olivera, Julián Felipe Porras-Villamil, Màrius Vicent Fuente

**Affiliations:** 1 Grupo de Parasitología, Instituto Nacional de Salud, Bogotá, D. C., Colombia Grupo de Parasitología Instituto Nacional de Salud Bogotá, D. C. Colombia; 2 Grupo de Investigación Parásitos y Salud, Facultat de Farmacia i Ciencies de l’Alimentació, Universitat de Valencia, Valencia, España Universitat de Valencia Grupo de Investigación Parásitos y Salud Facultat de Farmacia i Ciencies de l’Alimentació Universitat de Valencia Valencia Spain; 3 Facultad de Ciencias de la Salud, Universidad de la Salle, Bogotá, D. C., Colombia Universidad de la Salle Facultad de Ciencias de la Salud Universidad de la Salle Bogotá, D. C. Colombia

**Keywords:** Ascaris lumbricoides, Trichuris, climate change, helminthiasis, helminths, public health, Ascaris lumbricoides, Trichuris, cambio climático, helmintiasis, helmintos, salud pública

## Abstract

**Introduction.:**

Soil-transmitted helminthiases remain a significant public health burden in Colombia, especially in rural and tropical areas. Climate change is expected to alter environmental conditions that favor the survival and transmission of *Ascaris lumbricoides*, *Trichuris trichiura*, and hookworms.

**Objective.:**

To estimate the current spatial distribution of these infections and project prevalence changes by 2035 under climate change scenarios, with and without public health interventions.

**Materials and methods.:**

An ecological study with spatial modeling was conducted, integrating epidemiological, climatic, and biological data. Baseline prevalence data were obtained from the *Encuesta Nacional de Parasitismo Intestinal* (2012-2014). Climate projections from the ERA5-Land satellite product (2024-2035) were used alongside generalized additive models to estimate environmental suitability. A systematic review defined optimal temperature and humidity thresholds for the development of infective stages. Two scenarios were modeled: one without intervention and another with mass drug administration and improved sanitation.

**Results.:**

Baseline prevalence was 11.3% for *A. lumbricoides*, 18.4% for *T. trichiura*, and 6.4% for hookworms, with highest rates in Amazonia and the Sierra Nevada de Santa Marta. In a no-intervention scenario, projected prevalences increased to 13.6, 21.2, and 8.0%, respectively. The intervention scenario reduced these to 6.8%, 12.7%, and 5.6%. Temperature and humidity were strong positive predictors (p < 0.01), while altitude and forest cover showed negative associations.

**Conclusions.:**

Climate change may intensify soil-transmitted helminthiases transmission in Colombia by 2035. However, sustained control strategies could significantly mitigate this impact. Spatial modeling offers a valuable tool to guide targeted interventions and inform public health planning.

## Resumen

**Introducción.:**

Las geohelmintiasis siguen siendo una carga importante para la salud pública en Colombia, especialmente en zonas rurales y tropicales. Se espera que el cambio climático modifique las condiciones ambientales que favorecen la supervivencia y la transmisión de *Ascaris lumbricoides*, *Trichuris trichiura* y uncinarias.

**Objetivo.:**

Estimar la distribución espacial actual de estas infecciones y proyectar cambios en la prevalencia hacia 2035 con el cambio climático, con intervenciones en salud pública y sin ellas.

**Materiales y métodos.:**

Se realizó un estudio ecológico con modelado espacial que integró datos epidemiológicos, climáticos y biológicos. La prevalencia basal provino de la Encuesta Nacional de Parasitismo Intestinal (2012-2014). Se utilizaron proyecciones climáticas del producto satelital ERA5-Land (2024-2035) y modelos aditivos generalizados para estimar la idoneidad ambiental. Se establecieron rangos óptimos de temperatura y humedad mediante una revisión sistemática. Se modelaron dos escenarios: sin intervención y con desparasitación masiva y mejoras en saneamiento.

**Resultados.:**

La prevalencia basal fue de 11,3 % para *A. lumbricoides*, 18,4 % para *T. trichiura* y 6,4 % para uncinarias, con tasas más altas en la Amazonía y la Sierra Nevada de Santa Marta. Sin intervención, las prevalencias proyectadas aumentaron a 13,6, 21,2 y 8,0 %, respectivamente. Con intervención, se redujeron a 6,8, 12,7 y 5,6 %. La temperatura y humedad fueron predictores positivos significativos (p < 0,01), mientras que la altitud y la cobertura boscosa mostraron asociaciones negativas.

**Conclusiones.:**

El cambio climático podría intensificar la transmisión de las geohelmintiasis en Colombia hacia el 2035, pero las estrategias sostenidas de control pueden mitigar este impacto. El modelado espacial es una herramienta útil para planificar intervenciones focalizadas en salud pública.

Soil-transmitted helminthiases remain one of the most significant public health problems worldwide, particularly in low- and middle-income regions. It is estimated that over 1.5 billion people are infected with one or more intestinal nematodes (*Ascaris lumbricoides*, *Trichuris trichiura*, and hookworms), resulting in direct consequences for growth, cognitive development, and school performance in children, as well as indirect effects on productivity and quality of life ^1^^,^^2^. Although the global burden has declined over the past decade due to mass deworming interventions ^3^, transmission persists in settings of structural poverty, lack of basic sanitation, and limited access to healthcare services ^4^.

In Colombia, soil-transmitted helminthiases primarily affect children in rural, indigenous, and peripheral areas. Recent studies have reported concerning prevalence rates in regions with limited access to safe drinking water and proper sanitation ^5^^,^^6^. Despite national efforts to improve control, significant gaps remain in the precise identification of high-risk areas and in the territorial planning of targeted interventions ^7^. This situation is exacerbated by the historically fragmented surveillance of these infections, which limits timely responses to environmental changes.

Climate change has emerged as a new challenge in the control of neglected tropical diseases. Shifts in temperature, humidity, and precipitation may alter the spatial distribution of parasites, their vectors, or their environmental stages, facilitating expansion into new areas or resurgence in previously controlled regions ^8^^,^^9^. For soil-transmitted helminthiases, the biological cycle includes egg and larval stages in the environment, whose viability is critically dependent on soil and climatic conditions ^10^. Rising temperatures may accelerate larval development, while humidity and rainfall affect the survival and dispersion of infective stages ^11^^,^^12^. These changes, in combination with social vulnerability patterns, could significantly reshape transmission circumstances ^13^.

Several studies have demonstrated the utility of spatial and climate-based models to anticipate the geographic distribution of parasitic diseases under current and future climate changes ^14^^,^^15^. Such approaches allow for the identification of potentially affected areas, prioritization of resources, and strategic planning of surveillance and control efforts based on environmental and epidemiological dynamics. In Colombia, detailed projections for soil- transmitted helminthiases under climate change conditions are still lacking, representing a critical gap in the design of sustainable interventions.

This study aims to estimate the current spatial distribution of soil- transmitted helminthiases in Colombia and to project changes in risk areas by 2035 under climate change conditions. The findings are intended to inform public health policies, territorial planning, and elimination strategies.

## Materials and methods

### Study design and spatial modeling

An ecological spatial modeling study was conducted to estimate the current and projected distribution of soil-transmitted helminthiases in Colombia under different climate change conditions. Epidemiological data on the presence of *A. lumbricoides*, *T. trichiura*, and hookworms were used, along with historical and future environmental and climatic variables obtained from official sources and global databases. The analysis applied spatial modeling approaches to delineate regions with ecological and climatic conditions favorable for the transmission of soil-transmitted helminthiases.

### Systematic review for biological parameterization

To establish the physiological limits for survival and development of infective stages in the environment, a systematic review of the scientific literature was conducted following PRISMA (Preferred Reporting Items for Systematic Reviews and Meta-Analyses) guidelines. The search was carried out in PubMed, Scopus, and Web of Science using combinations of terms related to temperature, humidity, soil-transmitted helminthiases, and free- living stages (*e.g.*, egg, larvae, survival, development). Studies conducted under laboratory conditions with controlled environmental variables were included if they reported quantitative data on egg or larval development and survival under specific climatic conditions. A total of 7 studies met the inclusion criteria after screening 153 records (supplementary file 1).

Extracted temperature and humidity ranges were compiled into a comparative matrix and used as ecological filters in the modeling process. Additionally, development and survival rates were calculated as the inverse of median times (days^-1^) reported in the studies. Egg mortality rate (μ_e_) was estimated using the formula: μ_e_ = (M x h) / (1 - M), where M is the proportion of unhatched eggs and h the hatching rate.

### Data sources and extracted variables

Multiple sources of epidemiological, climatic, and geospatial information were integrated. Prevalence data were obtained from the *Encuesta Nacional de Parasitismo Intestinal* (2012-2014) in school-aged children, which provides representative data at municipal and departmental levels ^5^. Climate projections for 2024-2035 were sourced from the *Instituto de Hidrología, Meteorología y Estudios Ambientales* (IDEAM) and adjusted by hydrological region. These include projected changes in maximum, minimum, and mean temperature and precipitation, aligned with Intergovernmental Panel on Climate Change (IPCC) representative concentration pathways. Historical climatic data (2010-2023) from ERA5-Land were accessed through Google Earth Engine and aggregated at weekly scale. Precipitation averages and projected variations were combined to simulate future conditions. Land cover and land-use changes were derived from MapBiomas Colombia.

### Spatial analysis and data processing

Prevalence of soil-transmitted helminthiases was estimated by biogeographic province based on the proportion of infected schoolchildren, aggregated at the municipal and provincial levels ^16^. This ecological classification reflects Colombia’s environmental heterogeneity and includes the following regions:

- Caribbean oceanic islands,
- Pacific oceanic islands,
- Pericaribbean arid belt,
- Sierra Nevada de Santa Marta,
- Chocó-Magdalena,
- Orinoquia,
- Guayana,
- VIII.Amazonia, and
- Norandina

The spatial database was constructed using Microsoft Excel (Microsoft Corp., Redmond, WA, USA) to organize epidemiological and environmental data. Geographic visualizations and thematic maps were generated with QGIS software, enabling the representation of prevalence patterns across diverse ecological regions.

To assess the influence of environmental variables on soil-transmitted helminthiases transmission, generalized additive models were applied in R. These models account for non-linear relationships between predictors (temperature, humidity, altitude, land cover) and prevalence using smoothing functions. Ecological limits derived from the systematic review were incorporated as covariates. Model selection was based on minimization of generalized cross-validation score and evaluation of residual plots.

### Model validation and sensitivity analysis

Model performance was assessed using the deviance explained, adjusted R^2^, and Akaike information criterion. Cross-validation was conducted using k-fold (k = 5) procedures to test the robustness of predictions. Sensitivity analyses were performed by varying ecological thresholds (World Health Organization ± 10%) and comparing shifts in suitability maps to evaluate model stability under parameter uncertainty.

### Conditions modeling with and without intervention

In addition to the baseline projection (without intervention), a second scenario incorporated mass drug administration and sanitation improvements. Based on World Health Organization (WHO) guidelines and published evidence, the model applied reductions of 50% for *A. lumbricoides*, 40% for *T. trichiura*, and 30% for hookworms by 2035. These reductions were applied to the no-intervention scenario to estimate the potential impact of sustained control efforts. Differences were visualized through comparative maps and bar plots, with 95% confidence intervals (95% CI).

### Ethical considerations

This study did not involve direct participation of human subjects or the use of identifiable personal data and was therefore classified as minimal-risk research, in accordance with Resolution 8430 of 1993 from the Colombian *Ministerio de Salud y Protección Social*. All data used was publicly available or anonymized, and confidentiality was ensured through the use of deidentified datasets. Accordingly, approval from an ethics committee was not required. The study was conducted in compliance with the ethical principles of the Declaration of Helsinki.

## Results

### Baseline prevalence of soil-transmitted helminthiases infections in Colombia

According to data from the *Encuesta Nacional de Parasitismo Intestinal* (2012-2014), the estimated national prevalence was 11.3% for *A. lumbricoides*, 18.4% for *T. trichiura*, and 6.4% for hookworms. The highest prevalences were recorded in the Sierra Nevada de Santa Marta and the Amazonia region, where *A. lumbricoides* reached 42.9 and 58.0%, respectively; *T. trichiura*, 61.0% and 50.0%; and hookworms, 3.4 and 35.7%. The Orinoquia, Guayana, and Norandina regions showed low prevalences of *A. lumbricoides* and *T. trichiura*, but moderate prevalence of hookworms. These findings reveal a heterogeneous geographic distribution of parasitic burden among schoolchildren, influenced by ecological and socioeconomic factors (table 1).

**Table 1:** Baseline prevalence (%) of *Ascaris lumbricoides*, *Trichuris trichiura*, and hookworms by biogeographic province in Colombia, 2012-2014

Biogeographic region	***Ascaris lumbricoides* (%)**	95% CI	***Trichuris trichiura* (%)**	IC 95%	Hookworms (%)	95% CI
Insular Caribbean	2.0	1.2 - 3.8	9.8	6.9 - 13.5	0.0	0.0 - 0.2
Arid pericaribbean belt	20.1	16.5 - 24.7	42.7	37.9 - 47.8	13.2	10.1 - 17.0
Sierra Nevada de Santa Marta	42.9	37.8 - 48.1	61.0	56.0 - 66.0	3.4	2.1 - 5.6
Chocó-Magdalena	16.7	13.1 - 21.0	30.0	25.3 - 35.1	8.9	6.3 - 12.3
Orinoquia	0.2	0.1 - 0.5	14.5	11.2 - 18.5	9.8	7.2 - 13.2
Guayana	0.6	0.3 - 1.2	4.8	3.1 - 7.4	10.8	7.9 - 14.6
Amazonia	58.0	51.3 - 64.2	50.0	44.6 - 55.7	35.7	30.1 - 41.9
Norandina	3.2	2.1 - 4.9	4.3	3.0 - 6.0	1.0	0.5 - 2.0
Countrywide	11.3	10.2 - 12.6	18.4	16.9 - 19.9	6.4	5.6 - 7.3

**Figure 1:**
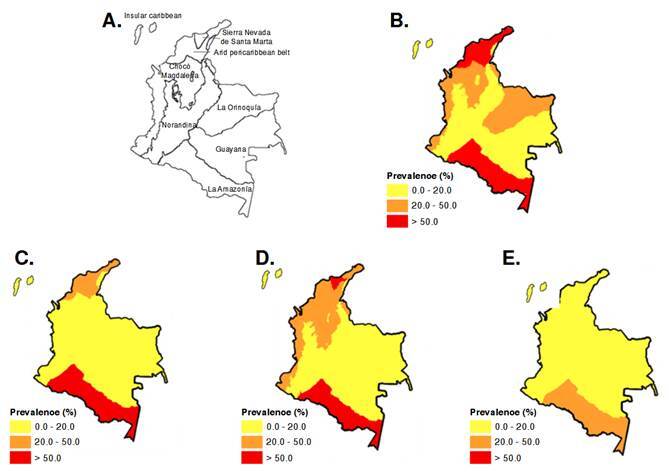
Baseline geographic distribution of soil-transmitted helminthiases prevalence by biogeographic regions. **A.** Biogeographic region. **B.** Aggregated national map of geohelminth prevalence **C.** *Ascaris lumbricoides* **D.** *Trichuris trichiura* **E.** Hookworms

The spatial distribution of prevalence by biogeographic site is shown in species-specific maps, highlighting the geographic heterogeneity of the parasitic burden across the national territory (figure 1A-1E). Figure 1B displays the aggregated national baseline prevalence for the soil-transmitted helminthiases, positioning the Sierra Nevada de Santa Marta and Amazonia as critical transmission hotspots.

### Prevalence projections for 2035 under climate change conditions

Under a projected climate change scenario without intervention, a generalized increase in soil-transmitted helminthiases prevalence by 2035 was estimated. Based on average increases reported in the literature (20% for *A. lumbricoides*, 15% for *T. trichiura*, and 25% for hookworms), national prevalences were projected at 13.6%, 21.2%, and 8.0%, respectively. The locations with the highest projected increases were the Sierra Nevada de Santa Marta and Amazonia. In the latter, projected prevalences were 69.6% for *A. lumbricoides*, 57.5% for *T. trichiura*, and 44.6% for hookworms. These estimates reflect a high-risk condition in the absence of control measures and underscore the vulnerability of humid and tropical regions (table 2).

**Table 2:** Projected prevalence (%) of soil-transmitted helminthiases by biogeographic region in 2035 under a climate change scenario without intervention

Biogeographic region	***Ascaris lumbricoides* (%)**	95% CI	***Trichuris trichiura* (%)**	IC 95%	Hookworms (%)	95% CI
Insular Caribbean	2.3	1.4 - 4.1	11.3	8.1 - 15.6	0.1	0.0 - 0.3
Arid pericaribbean belt	24.5	20.3 - 29.3	49.1	43.0 - 54.9	16.5	12.5 - 21.3
Sierra Nevada de Santa Marta	51.5	45.7 - 58.2	70.2	64.2 - 76.1	4.1	2.4 - 6.7
Chocó-Magdalena	19.8	15.9 - 24.7	34.5	29.2 - 40.3	10.7	7.6 - 14.7
Orinoquia	0.3	0.1 - 0.6	16.7	13.0 - 21.1	11.9	8.6 - 16.1
Guayana	0.7	0.4 - 1.3	5.4	3.5 - 8.2	12.7	9.1 - 17.3
Amazonia	69.6	62.6 - 76.6	57.5	51.4 - 63.6	44.6	40.1 - 49.1
Norandina	3.8	2.4 - 6.1	5.2	3.5 - 7.4	1.3	0.7 - 2.4
Countrywide	13.6	12.1 - 15.3	21.2	19.6 - 23.1	8.0	7.0 - 9.1

These projections indicated variability between locations. For example, in Amazonia, the projected prevalence of *A. lumbricoides* was 69.6% (95% CI: 62.6-76.6), and for hookworms, 44.6% (95% CI: 40.1-49.1).

**Figure 2:**
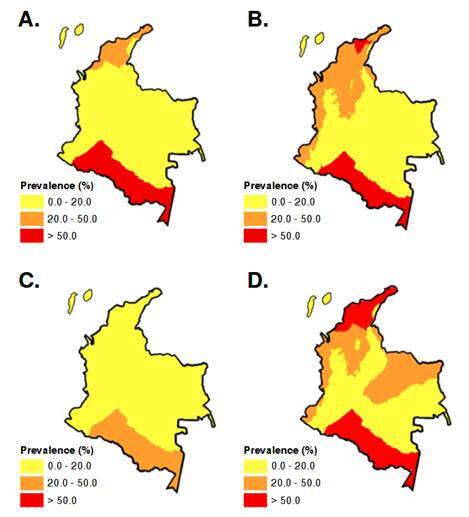
Projected prevalence maps of soil-transmitted helminthiases infections for the year 2035 under a climate change scenario without intervention. **A.** *Ascaris lumbricoides* **B.** *Trihuris trichiura* **C.** Hookworms **D.** Aggregated national map of projected prevalence.

Spatial projections for 2035 under the no-intervention scenario are presented in the corresponding maps (figure 2A-2C), showing a generalized increase in warm and humid areas such as Amazonia, Chocó-Magdalena, and the Sierra Nevada de Santa Marta. Figure 2D presents the aggregated national distribution projected under this scenario.

### Intervention conditions: antiparasitic treatment and sanitation improvement

**Figure 3:**
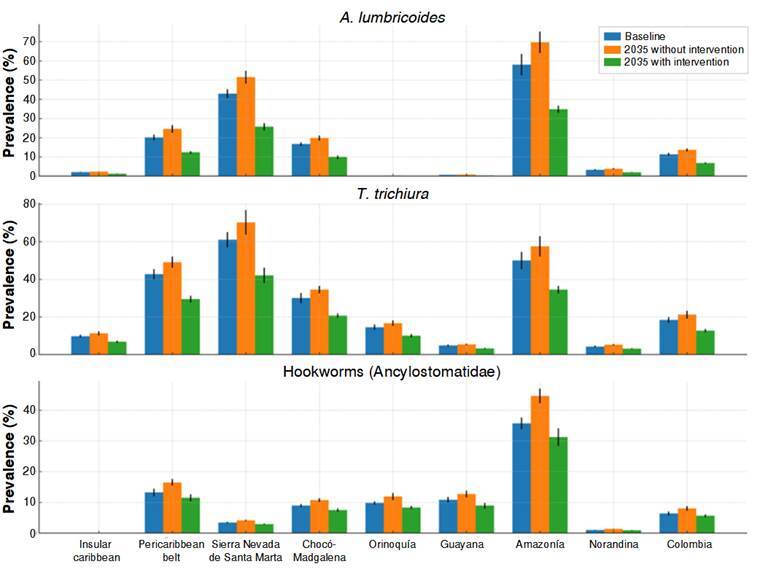
Comparison of projected soil-transmitted helminthiases prevalence in 2035 under no-intervention and intervention scenarios

In response to the projected scenario, an intervention model was explored based on WHO-recommended strategies. An alternative scenario was modeled, including mass deworming and progressive improvement in sanitation access, assuming reductions of 50% in projected prevalence for *A. lumbricoides*, 40% for *T. trichiura*, and 30% for hookworms. Results show a substantial mitigating effect across all regions, particularly in Amazonia and Chocó-Magdalena. Adjusted projections are presented in table 3, and the integrated comparison in figure 3 demonstrates a clear reduction in parasitic burden under this circumstance. This suggests that sustained intervention could partially offset the adverse effects of climate change on transmission. This suggests that sustained intervention, even in complex ecological settings such as Amazonia and the Sierra Nevada, could partially offset the adverse effects of climate change on transmission.

**Table 3:** Projected prevalence (%) of soil-transmitted helminthiases in 2035 under an intervention scenario with mass drug administration and improved sanitation

Biogeographic region	***Ascaris lumbricoides* (%)**	95% CI	***Trichuris trichiura* (%)**	IC 95%	Hookworms (%)	95% CI
Insular Caribbean	1.2	0.7 - 2.3	6.8	4.5 - 10.2	0.07	0.0 - 0.2
Arid pericaribbean belt	12.3	9.9 - 15.6	29.5	24.3 - 35.3	11.5	8.4 - 15.4
Sierra Nevada de Santa Marta	25.7	21.8 - 30.5	42.1	36.5 - 48.2	2.9	1.6 - 5.1
Chocó-Magdalena	9.9	7.2 - 13.6	20.7	16.2 - 25.9	7.5	5.1 - 10.8
Orinoquia	0.15	0.05 - 0.3	10.0	7.0 - 13.5	8.3	5.8 - 11.9
Guayana	0.35	0.2 - 0.6	3.2	2.0 - 5.1	8.9	6.2 - 12.7
Amazonia	34.8	29.6 - 40.3	34.5	29.3 - 40.1	31.2	26.3 - 36.7
Norandina	1.9	1.1 - 3.4	3.1	2.0 - 4.8	0.9	0.4 - 1.8
Countrywide	6.8	6.0 - 7.8	12.7	11.2 - 14.3	5.6	4.8 - 6.5

**Figure 4:**
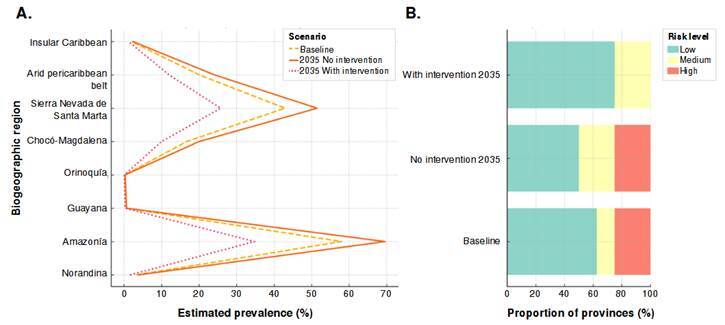
Evolution of *Ascaris lumbricoides* infection risk across biogeographic provinces under different scenarios: **A.** Prevalence estimates for 2035 **B.** Change in number of provinces classified as high-risk areas.

Figure 4 provides a visual summary of the projected evolution of *A. lumbricoides* infection across the modeled scenarios. Panel A shows a widespread increase in estimated prevalence in most biogeographic areas by 2035 without intervention, with a marked reduction when a control condition based on treatment and sanitation improvements is included. Panel B illustrates how the proportion of areas classified as high-risk shifts from 25% under current conditions to 50% without intervention but decreases to 12.5% with intervention.

### Environmental factors associated with transmission

Multivariate analysis using generalized additive models revealed statistically significant associations between environmental variables and the prevalence of all three soil-transmitted helminthiases species. Mean temperature showed a consistent positive association with the prevalence of *A. lumbricoides* (β = 0.18; 95% CI: 0.06-0.31; p = 0.004), *T. trichiura* (β = 0.22; 95% CI: 0.09-0.35; p = 0.001), and hookworms (β = 0.30; 95% CI: 0.110.49; p = 0.002).

Similarly, relative humidity had a significant positive effect in all models, with the greatest impact on hookworms (β = 0.20; 95% CI: 0.05-0.34; p = 0.008). In contrast, altitude was negatively associated with the prevalence of *A. lumbricoides* (β = -0.12; 95% CI: -0.23 to -0.01; p = 0.032) and *T. trichiura* (P = -0.17; 95% CI: -0.29 to -0.05; p = 0.008), while forest cover showed a mild but significant inhibitory effect for hookworms (β = -0.09; 95% CI: -0.17 to -0.01; p = 0.042).

**Figure 5:**
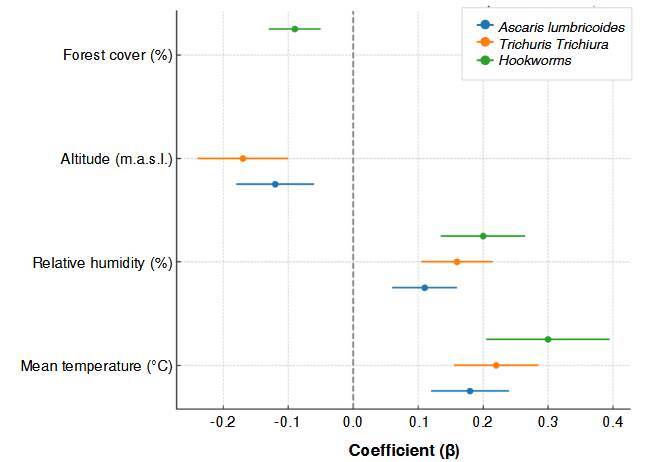
Associations between environmental predictors and soil-transmitted helminthiases prevalence from generalized additive models

These findings confirm that temperature and humidity are key environmental determinants of soil-transmitted helminthiases transmission and support their inclusion as core predictors in projected spatial scenarios (table 4, figure 5).

**Table 4:** Environmental predictors of soil-transmitted helminthiases prevalence identified through generalized additive models

Environmental variable	Helminth	Coefficient (βi)	95% CI	p value
Mean temperature (°C)	*Ascaris lumbricoides*	0.18	0.06 - 0.31	0.004
Mean temperature (°C)	*Trichuris trichiura*	0.22	0.09 - 0.35	0.001
Mean temperature (°C)	Hookworms	0.30	0.11 - 0.49	0.002
Relative humidity (%)	*Ascaris lumbricoides*	0.11	0.01 - 0.21	0.029
Relative humidity (%)	*Trichuris trichiura*	0.16	0.05 - 0.27	0.006
Relative humidity (%)	Hookworms	0.20	0.05 - 0.34	0.008
Altitude (m.a.s.l.)	*Ascaris lumbricoides*	- 0.12	-0.23 to -0.01	0.032
Altitude (m.a.s.l.)	*Trichuris trichiura*	- 0.17	-0.29 to -0.05	0.008
Forest cover (%)	Hookworms	- 0.09	-0.17 to -0.01	0.042

### Optimal environmental conditions and infective forms

**Figure 6:**
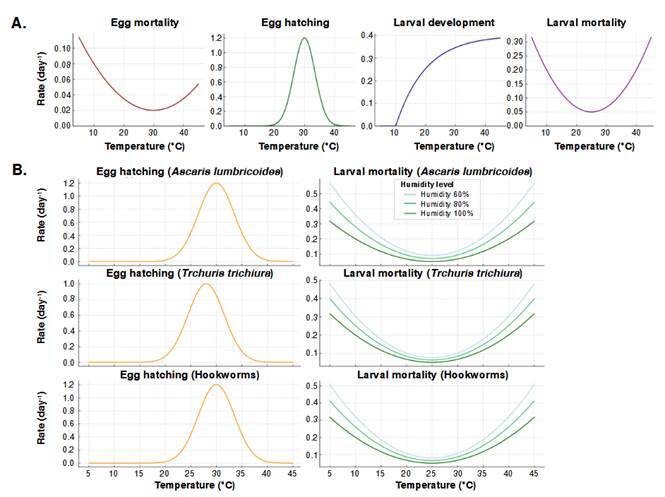
Optimal environmental conditions for the infective stages of soil-transmitted helminthiases (*Ascaris lumbricoides*, *Trichuris trichiura*, and hookworms). **A.** Relationship between temperature and biological parameters **B.** Comparative effects of temperature and humidity

The systematic review identified the most favorable thermal and humidity ranges for the development of infective stages of soil-transmitted helminthiases (table 5, figure 6). for *A. lumbricoides*, the optimal temperature range for development was 28-32 °C, with relative humidity ≥ 80%. For *T. trichiura*, optimal values were slightly lower: 26-30 °C and ≥ 70% humidity. Hookworms (*Ancylostoma* spp. and *Necator americanus*) showed a thermal range similar to *A. lumbricoides* (27-32 °C) and require relative humidity ≥ 80% for effective larval development.

**Table 5:** Optimal temperature and humidity ranges for the development of infective stages of *Ascaris lumbricoides*, *Trichuris trichiura*, and hookworms, based on a systematic review

Helminth	Evaluated stage	Optimal temperature range (°C)	Minimum threshold (°C)	Maximum threshold (°C)	Optimal relative humidity (%)	Estimated development time
*Ascaris lumbricoides*	Embryonated eggs	28 - 32	15	38	≥ 80	2 - 5 weeks
*Trichuris trichiura*	Embryonated eggs	26 - 30	10	35	≥ 70	2 - 5 weeks
*Ancylostoma duodenale*	Larvae (L_1_-L_3_)	25 - 30	14	37	≥ 85	5 - 10 days
*Necator americanus*	Larvae (L_1_-L_3_)	27 - 32	17	40	≥ 80	5 - 10 days

Extreme thresholds were also documented, with minimum temperatures between 10 and15 °C and maximums between 35 and 40 °C. These define the ecological limits beyond which environmental development of parasites cannot occur.

Regarding infective forms, both *A. lumbricoides* and *T. trichiura* reach their infective stage as embryonated eggs, which require 2 to 5 weeks to mature under optimal moist and warm soil conditions. In contrast, hookworms develop into infective filariform larvae (L_3_) within a shorter period, 5 to 10 days, which may provide an adaptive advantage in favorable environments.

These biological parameters, extracted from a systematic review of experimental studies, were used as ecological restriction criteria in the spatial models and were key to interpreting the current and projected distribution of these species.

## Discussion

This study integrated epidemiological, environmental, and biological data to model the current and future distribution of intestinal soil-transmitted helminthiases in Colombia using a spatial and climate-sensitive approach. The findings reveal a highly heterogeneous transmission pattern, shaped by geographic, ecological, and social factors, with the Sierra Nevada de Santa Marta and the Amazonia region emerging as hyperendemic zones. This distribution aligns with areas of high rainfall, sustained humidity, and dense forest cover, suggesting that environmental determinants play a key role in the persistence and expansion of transmission ^2^^,^^13^^,^^17^.

Projections for 2035 under a no-intervention scenario indicate a significant increase in the prevalence of *A. lumbricoides*, *T. trichiura*, and hookworms. These estimates are consistent with studies from other tropical regions such as southeast Asia and sub-Saharan Africa, where climate change has been linked to altitudinal and latitudinal expansion of parasitic diseases ^18^^-^^20^. However, few studies in Latin America have assessed this phenomenon with a spatial and species-specific perspective, highlighting the relevance of the present analysis. The most vulnerable areas were those where current environmental conditions already fall within the optimal ranges for the development of infective stages ^21^^,^^22^.

The analysis using generalized additive models identified temperature and relative humidity as the most relevant predictors for all three species, supporting earlier findings ^10^^,^^13^ that described nonlinear relationships between temperature, precipitation, and soil-transmitted helminthiases prevalence. The negative association with altitude and the moderate inhibitory effect of forest cover have also been observed in studies from Ethiopia and southeast Asia ^23^^,^^24^. In this study, however, the effects varied by species, suggesting that the ecological niche of each soil-transmitted helminthiases may be modulated by micro-environmental variations and host-related factors ^25^.

The systematic review contributed to the biological validation of the spatial projections by identifying the temperature and humidity thresholds that define environmental suitability for each species. This approach strengthened the model’s robustness by incorporating ecological constraints based on experimental evidence ^26^^,^^27^. Unlike models based solely on empirical correlations, this study integrated detailed parasitological knowledge, allowing for a more accurate interpretation of distribution and environmental persistence. In this sense, differences between *A. lumbricoides* and hookworms in terms of thermal sensitivity and moisture requirements help explain their uneven geographic distribution across Colombia ^28^^,^^29^.

The characterization of infective forms also provides critical insights for planning interventions. The fact that *Ascaris* spp. and *Trichuris* spp. eggs require several weeks to mature, while hookworm larvae reach the infective stage in less than 10 days, implies that extreme climatic events (e.g., heavy rains or sudden temperature increases) could differentially affect transmission dynamics between species ^30^^-^^32^. This reinforces the need to account for biological differences when designing targeted control strategies, especially in regions where multiple species coexist with divergent life cycles ^33^.

In addition to the no-intervention scenario, this study modeled for the first time in Colombia an alternative scenario incorporating school-based mass deworming and progressive improvements in sanitation. Under this projection, estimated prevalence by 2035 was reduced by up to 50% for *Ascaris*, 40% for *Trichuris*, and 30% for hookworms. These results are consistent with soil- transmitted helminthiases control impact studies in Brazil, Cambodia, and Ethiopia, where the combination of periodic treatment and improved basic services significantly reduced parasitic burden ^34^^-^^36^. This comparison highlights the mitigating potential of public health policies in counteracting the amplifying effects of climate change.

From a public policy perspective, the findings of this study help identify priority regions where climatic and ecological conditions favor persistent soil-transmitted helminthiases transmission. This evidence can inform costeffective interventions, such as school deworming campaigns targeting high-burden areas like the Amazonia and the Sierra Nevada de Santa Marta, or the promotion of basic sanitation infrastructure in municipalities with high projected environmental suitability.

Furthermore, the spatial visualization of risk helps raise awareness among decision-makers about the indirect health impacts of climate change on children. In remote and underserved areas, such as indigenous communities in the Amazonia and Sierra Nevada regions, implementing interventions like periodic deworming or improved sanitation infrastructure poses operational and financial challenges. However, successful pilot programs in Brazil and Perú have demonstrated the feasibility of school-based treatment through community health workers and intercultural health models adapted to local contexts. These experiences suggest that, with political will and adequate investment, targeted interventions are both achievable and impactful, especially when integrated into broader primary healthcare and educational frameworks. Spatial models such as those developed in this study can assist in prioritizing efforts, identifying high-risk Indigenous territories, and supporting culturally sensitive implementation plans.

This study presents some limitations that should be considered when interpreting the results. First, the baseline prevalence data corresponds to the 2012-2014 period. However, this data comes from the latest national survey with subnational representativeness, making them valid inputs for spatial analysis and prospective modeling. Despite the temporal gap, they reflect structural and persistent epidemiological patterns in regions with ecological conditions conducive to transmission. Second, the model did not incorporate sociodemographic variables such as population density, healthcare access, or hygiene practices, which can alter real exposure risk. Third, *Strongyloides stercoralis* was excluded from the analysis due to its distinct biological cycle, which is less dependent on climatic conditions. In addition, commonly used diagnostic methods have low sensitivity for its detection, geospatial data are limited, and the study focused on priority species for mass control intervention. Furthermore, projections were based on a single climate scenario and did not account for broader mitigation strategies or alternative emissions trajectories. Lastly, although official prevalence data was used, they may not reflect emerging foci or underreporting in remote areas.

Despite these limitations, the study presents several methodological strengths. These include the integration of diverse data sources - epidemiological, environmental, biogeographic, and climatic- the use of non-linear models that capture complex relationships among variables, and the incorporation of a systematic review to parameterize the environmental behavior of soil-transmitted helminthiases. This combination of approaches provides a robust analytical framework and offers a practical tool for early warning surveillance, spatial planning, and the design of context-specific interventions tailored to Colombia’s ecological and climatic landscape.

In conclusion, this study provides robust evidence on how climate change could intensify the transmission of soil-transmitted helminthiases in Colombia, particularly in humid regions such as the Amazonia and the Sierra Nevada de Santa Marta. By integrating epidemiological, climatic, and biological data, high-precision risk areas were identified using non-linear models parameterized with experimental evidence.

Projections for 2035 under a no-intervention scenario indicate a significant increase in the prevalence of *A. lumbricoides*, *T. trichiura*, and hookworms. However, the control scenarios demonstrated that much of this impact could be mitigated through mass deworming and improvements in basic sanitation.

These findings provide a valuable foundation for guiding targeted public health policies and replicating this approach in other tropical settings, thereby strengthening territorial planning and health system resilience in the face of climate change.

## Figures and Tables

**Figure S1:**
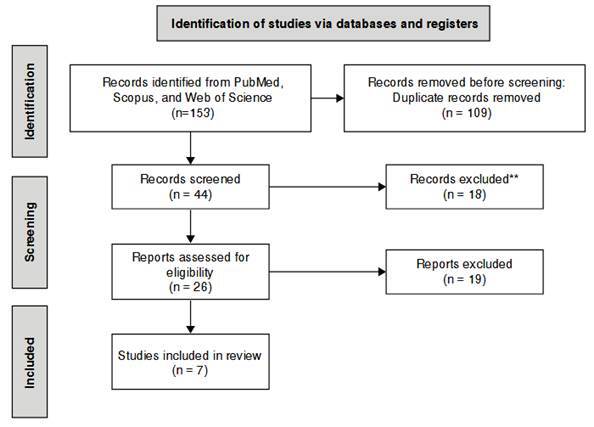
PRISMA flow diagram of study selection on climate effects on soil-transmitted helminths eggs and larvae

**Figure S2:**
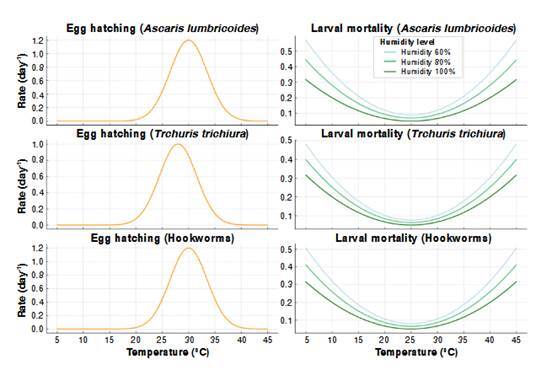
Relationship between temperature and biological parameters. B. Comparative effects of temperature and humidity.

**Figure S3:**
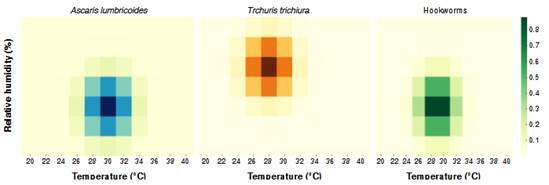
Temperature-soil-transmitted helminthiases interaction heatmap: Development and survival thresholds for human soil-transmitted helminthiases

**Figure S4:**
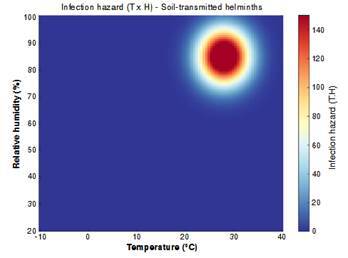
Hazard surface of soil-transmitted helminthiases infection as a function of relative humidity and temperature

**Figure S5:**
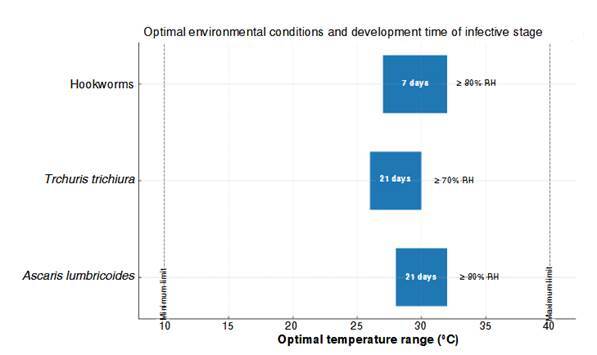
Optimal environmental conditions and development time of infective stage

## Supplementary files

Supplementary file 1. Spatial modelling of soil-transmitted helminth infections in Colombia under climate change scenarios

SI.1 Literature analysis

We followed the Preferred Reporting Items for Systematic Reviews and Meta-Analyses (PRISMA) guidelines to identify publications examining the effects of environmental factors on the survival and development of free-living stages of *Ascaris lumbricoides*, *Trichuris trichiura*, and hookworms (Ancylostomatoidea). The literature search focused on laboratory-based studies that evaluated how temperature and/or humidity influence the viability and development of parasite eggs or larvae.

We used the following keyword combination to conduct our search:

( ( ( "Temperature" OR "Humidity" ) AND ( "Ascaris" OR "Trichuris" OR "Ancylostomatoidea" ) AND ( "egg" OR "larvae" ) AND ( "survival" OR "development" ) ) )

A total of 153 publications were initially identified. We applied the following inclusion criteria:

- Studies must have been conducted under laboratory conditions with controlled environmental variables.
- The study must report associated temperature and/or humidity values in relation to helminth egg or larval development.
- The parasites evaluated must belong to soil-transmitted helminthiases infecting humans.

After removal of 109 duplicates, 44 studies were screened based on title and abstract. Eighteen were excluded at this stage for not meeting the inclusion criteria. The remaining 26 full-text articles were reviewed, and 7 studies met all criteria and were included in the final analysis (Figure S1).

SI.2 Statistical analysis

From the selected studies, we extracted the reported median times (in days) for egg hatching, larval development (L_1_-L_3_), and survival of infective stages under specific climatic conditions. We then calculated the respective development and survival rates as the reciprocal of the median time (in days^-1^). Egg mortality rate (μ_e_) was estimated based on the hatching rate (h) and the proportion of unhatched eggs (M) at the end of the experiment, using the formula: μ_e_ = (M × h) / (1 - M)
